# Integrated metabarcoding and culture-dependent assessments reveal *Pseudomonas* as dominant hyphosphere-pathobiont in Race 4 *Fusarium* wilt pathogen of cotton

**DOI:** 10.3389/fmicb.2025.1661556

**Published:** 2025-09-04

**Authors:** Sanjay Antony-Babu, Gayan Abeysinghe, Vanessa Elizabeth Thomas, Cara Hockenbury, Shravan Sharma Parunandi, Amrita Dasgupta, Tristan Andrew Gregory, Amrita Sai Gabu, Haden Ball, Thomas M. Chappell, Brian D. Shaw, Thomas Isakeit, Elizabeth A. Pierson

**Affiliations:** ^1^Department of Plant Pathology and Microbiology, Texas A&M University, College Station, TX, United States; ^2^Department of Horticulture, Texas A&M University, College Station, TX, United States

**Keywords:** *Fusarium* wilt, FOV4, hyphosphere-bacteria, pathobiome, bacterial genomes

## Abstract

The hyphosphere, the microhabitat surrounding fungal hyphae, hosts complex microbial interactions that can influence fungal biology, yet the microbial community in hyphospheres of pathogenic fungi are seldom characterized. In this study, we investigated the hyphosphere of *Fusarium oxysporum* f. sp. *vasinfectum* Race 4 (FOV4), a major fungal pathogen threatening cotton, to characterize its bacterial community and assess potential functional roles. An integrated approach was employed combining confocal time-lapse microscopy, 16S rRNA metabarcoding, culture-dependent bacterial isolation, whole genome sequencing, and fungal-bacterial coculture assays. Microscopy confirmed hyphosphere association, and the bacterial predisposition towards the growing hyphal tips. Metabarcoding showed a stable hyphosphere community dominated by a single *Pseudomonas* ASV accounting for over 95% of relative abundance, with strong negative correlations to most other taxa. To evaluate the functions, ten representative bacterial isolates were sequenced, revealing enrichment in metabolic pathways related to carbon, nitrogen, and sulfur cycling. In particular, *Pseudomonas laurylsulfatiphila* showed high counts of oxidoreductases and hydrolases. Coculture assays demonstrated that several bacterial isolates significantly promoted FOV4 hyphal extension, while having limited or inconsistent effects on other *Fusarium* strains, indicating strain-specific interactions. Together, the findings reveal a stable and functionally enriched bacterial community in the FOV4 hyphosphere, with potential implications for fungal fitness and virulence. These results support the emerging concept of a hyphosphere-pathobiome and highlight microbial associations as targets for future plant disease management strategies.

## 1 Introduction

Soil is a dynamic habitat where fungi and bacteria engage in intimate interactions, especially in the hyphosphere, the zone immediately surrounding the fungal hyphae (Pugh and Sewell, 1958; Frey-Klett et al., 2011; Sun et al., 2023). The hyphosphere has been recognized as a hotspot of inter-kingdom signaling and nutrient exchange, analogous to the plant rhizosphere but centered on fungal mycelia (Steffan et al., 2020; Faghihinia et al., 2023). Fungal hyphae exude and emit diverse metabolites including soluble and volatile organic compounds (VOCs) that shape the microbial community in this zone (Abeysinghe et al., 2020), and conversely, hyphae-associated bacteria respond with their own signals and activities, leading to a complex chemical dialogue between kingdoms. Direct physical associations are common between these two partners, where the bacteria can attach to or move along hyphal surfaces, sometimes forming mixed-species biofilms (Frey-Klett et al., 2011; Antony-Babu et al., 2014; Deveau et al., 2016; Steffan et al., 2020). This “hyphal highway” phenomenon enables bacteria to disperse through soil by hitchhiking on fungal mycelium (Simon et al., 2015). Such dispersal not only benefits bacteria by granting access to new microsites and nutrients, but it may also influence fungal physiology through close proximity and molecular crosstalk. Indeed, myriad examples of fungal-bacterial cross-communication (via quorum-sensing molecules, VOCs, and other signals) have illustrated that the hyphosphere is a central arena for inter-kingdom interactions that can modulate the behavior and fitness of both partners (Garbaye, 1994; Frey-Klett et al., 2011).

Early work on fungal-bacterial relationships focused on bacteria associated with plant mutualistic fungi. In particular, certain soil bacteria have been termed “mycorrhiza helper bacteria” (or sometimes *fungal helper bacteria*) for their capacity to assist mycorrhizal fungi in establishing symbioses with plant roots (Garbaye, 1994; Frey-Klett and Garbaye, 2005; Bending, 2007; Tarkka and Frey-Klett, 2008; Frey-Klett et al., 2011). These helper bacteria often improve the physical or chemical environment for the fungus; for example, they may produce phytohormones that stimulate root exudation or antagonize other competing fungi, thereby facilitating mycorrhization (Frey-Klett and Garbaye, 2005; Bending, 2007). A well-known case is *Pseudomonas fluorescens* BBc6R8, which was shown to “prime” the growth and gene expression of the ectomycorrhizal fungus *Laccaria bicolor*, ultimately enhancing mycorrhizal establishment (Deveau et al., 2007; Cusano et al., 2011). Thus, the bacterial role in establishing fungal partnership with roots has been well established, albeit generally in the perspective of plant beneficial fungi.

The symbiotic relationships between plant-beneficial fungi and helper bacteria raise an intriguing question: do pathogenic fungi also form associations with bacteria to augment their fitness or pathogenic potential? Historically, research on fungal pathogens and bacteria emphasized antagonism (e.g., biocontrol bacteria that inhibit pathogens). However, growing evidence indicates that some pathogenic fungi form alliances with bacteria that can increase fungal pathogenicity. One striking example is the rice seedling blight fungus, *Rhizopus microsporus*, which harbors a *Burkholderia* endosymbiont that produces rhizoxin, the phytotoxin responsible for the disease lesions (Lackner et al., 2009). Similarly, *Rhizoctonia solani* was recently found to carry a dimorphic endofungal bacterium (*Enterobacter* sp.) that significantly enhances the fungus’ virulence on plants (Obasa et al., 2017). Obasa et al. (2020) demonstrated the presence of endohyphal *Enterobacter* sp. that enhances fumonisin production in *Fusarium fujikuroi* W343. Although studies of such pathogen-helper bacteria are still emerging, the concept of “fungal helper bacteria” now extends beyond mutualist-helpers to include pathogens. However, most of this evidence is from endohyphal bacteria, the bacteria that live within the fungal hypha (Moses and Carter, 2025). In natural systems, extrahyphal bacteria (those that exist in the hyphosphere) are more common, and require attention. Thomas and Antony-Babu (2024) recently demonstrated the existence of an extrahyphal bacteriome in the *Fusarium* wilt pathogen of watermelons. As freely moving organisms in the hyphosphere and along hyphal-highways, these bacteria represent biotic components that can be gained and lost in the environment. Together, the endohyphal and extrahyphal bacteria associated with pathogenic fungi likely contribute to plant diseases as proposed by the pathobiome concept (Thomas and Antony-Babu, 2024).

The pathobiome concept is a rapidly advancing understanding of pathosystems, where there is increased awareness that diseases must be examined beyond a one-pathogen one-disease paradigm. By definition, a pathobiome encompasses the pathogen incorporated within its biotic environment (Vayssier-Taussat et al., 2014; Bass et al., 2019). For soil-borne fungal pathogens, the hyphosphere bacteria represent a major biotic component that can affect their biology, similar to those that influence mycorrhizal fungal symbiosis. In the study presented here, we examine the hyphosphere-pathobiome of the *Fusarium* wilt pathogen of cotton (*Gossypium* spp.), termed *Fusarium oxysporum* f. sp. *vasinfectum* Race 4 (FOV4). This pathogen has been a major threat to cotton production, especially Pima cotton in Texas, USA (Halpern et al., 2017; Diaz et al., 2021; Davis et al., 2022; Wagner et al., 2022). Controlled inoculation and field studies have demonstrated that Race 4 can cause disease without prior root damage from wounding or nematodes (Halpern et al., 2017; Davis et al., 2022; Wagner et al., 2022), whereas race 1 typically requires nematode presence to induce disease. This characteristic raises the possibility that fungal-helper bacteria in the hyphosphere may assist the pathogen in disease development. As a first step towards this, we examined the hyphal-dwelling bacterial pathobionts of FOV4 from samples collected from El Paso County, Texas. In the study presented here, we demonstrate a stable bacterial community that occurs with the FOV4 strains, and the genetic potential of these hyphosphere bacteria towards enhancing/influencing FOV4 virulence.

## 2 Methods

### 2.1 Fungal pathogen isolation and inoculum preparation

A pathogenic isolate of *Fusarium oxysporum* f. sp. *vasinfectum* race 4 (FOV4) was recovered from symptomatic Pima cotton (*Gossypium barbadense*) plants exhibiting wilt symptoms in El Paso, Texas, USA (31.526674–106.193867). We labeled this isolate F4AB. The inoculum was prepared by combining 10 g of a growth matrix consisting of a 1:1 (w/w) sand:oatmeal (The Quaker Oat Bran™) mixture. This was dispensed into glass Petri plates and autoclaved for 20 min at 121 °C using the gravity cycle. Afterward, 200 μL of F4AB inoculum and 5 mL of 0.1x tryptic soy broth were added to the sand-oatmeal matrix. The cultures were then incubated at 27 °C for up to 7 days to facilitate growth. This culture acted as soil inoculum during hyphosphere bacteria cultivation.

Simultaneously, another inoculum was generated that contained FOV4 without any bacteria, either extra- or endo- hyphal bacteria. This cured FOV4 hyphae was obtained by treating the isolate with streptomycin (100 μg/mL) and tetracycline (20 μg/mL). The absence of the bacterial cells was confirmed with microscopy.

### 2.2 Hyphosphere bacterial cultivation

#### 2.2.1 Hyphosphere enrichment method

A custom soil matrix was prepared for the plant assay, consisting of 25% (v/v) field-collected cotton soil, 25% sifted commercial potting mix, and 50% play sand. Pima cotton seeds (“DP357”) were initially germinated on potting mix and later transplanted to the soil matrix. Approximately 1 week after germination, when the apical meristem had emerged, the fungal inoculum was introduced into each pot at a location approximately 2 cm from the seedling root zone to promote natural root colonization by the pathogen. Early wilting symptoms appeared approximately 2 weeks post-inoculation. Crown tissue from two symptomatic plants was collected at the soil interface and surface sterilized by sequential immersion in 10% bleach, 70% ethanol, and sterile distilled water for 2 min each. Sterile forceps were used to transfer the sterilized tissue onto half-strength potato dextrose agar (0.5x PDA) plates. Antibiotics were omitted to allow for the co-isolation of hyphae-associated bacteria. The plates were incubated at 27 °C for 4–5 days, ensuring fungal colonies did not reach the plate edges. These “*generation 1*” plates were used as inoculum for subsequent inoculations.

Aerial hyphae from generation 1 plates were carefully harvested using sterile toothpicks under a Plugable USB Digital Microscope (250 × magnification), avoiding contact with the agar surface. The hyphae, along with any associated bacteria, were suspended in 500 μL of molecular-grade water (5 PRIME, Germany) for downstream applications. For subsequent generations, 10 μL of the inoculum was placed at the center of 3 replicate fresh 0.5x PDA plates, creating “*generation 2*” plates. After 5 days, the colony edge was collected and transferred to molecular-grade water, as with the earlier generation. A triplicate for each of the 3 replicates was carried out as “*generation 3*.” This process resulted in 2 lineages of *generation 1* plates, with each plate serving as inoculum for 3 new plates, yielding a total of 26 replicate plates representing the hyphosphere of FOV4 across both generations and lineages, enabling comparative analysis of community stability.

#### 2.2.2 Microscopy

To confirm the presence of hyphosphere bacteria, time-lapse imaging was performed using a Fluoview FV300 confocal laser scanning microscope (Olympus) equipped with UAPON 100x OTIRF objective lens (numerical aperture 1.49). The samples were scanned in XYT mode with a spatial resolution of 1023×251 pixels (0.124 μm/pixel). Image acquisition was performed in a one-way scan at a sampling speed of 2.00 μs per pixel. A total of approximately 100 time points were acquired at 5 s intervals. Laser excitation was carried out using a 561 nm laser at 1.6% transmissivity with a DM405/488/561 dichroic mirror in place. Z-drift compensation was enabled during imaging. All images were acquired and processed using FV3000 system software.

### 2.3 Isolation of hyphosphere bacteria

The inoculum suspension from *generation 1*, which was used to prepare subsequent generation plates, was considered the main inoculum. Tenfold serial dilutions (10^−1^ to 10^−5^) of the inoculum were generated and plated onto MacConkey agar, yeast-extract mannitol agar, water yeast extract agar, oatmeal agar, Luria-Bertani agar, tryptic soy agar, and *Burkholderia mallei* agar. Tenfold serial dilutions were used to reduce bacterial density and enable the isolation of individual colonies, including slow-growing taxa. Multiple culture media were employed to maximize culturable diversity. All media were supplemented with nystatin (50 mg/mL) to inhibit F4AB growth. A part of the suspensions was heat-treated at 50 °C for 30 min to select for spore-forming bacteria, such as actinomycetes and *Bacillus* species. We envisaged that it is unlikely for bacteria to form spores while on the hypha, or even if they did, it would be unlikely we picked them from the aerial hypha. Hence, the inocula were enriched in tryptic soy broth for 48 h until we saw visible cell debris accumulating in the bottom of the culture tubes. Heat treatment was applied to these enrichments and plated on tryptic soy agar and oatmeal agar. Plates were incubated at 27 °C until colonies were observed.

### 2.4 Bacterial identification and whole genome sequencing

#### 2.4.1 16S rRNA based identification and hyphosphere community analysis

The 26 samples described in section 2.2.1 were aliquoted at 50 μL each, centrifuged, and the supernatant was discarded. The resulting pellets were resuspended in 1x TE buffer, followed by heat lysis at 95 °C for 10 min. The lysates were then centrifuged, and the supernatant containing the soluble cellular material, including DNA, was sent for sequencing at Novogene. Due to the limited biomass obtained, traditional DNA extraction methods were not feasible. The samples were used to generate V3–V4 16S rRNA gene libraries using the primers 341F (5’-CCTAYGGGRBGCASCAG-3′) and 806R (5’-GGACTACNNGGGTATCTAAT-3′), and sequencing was performed using the Illumina NovaSeq PE250 platform. Metabarcoding sequence analysis was processed in Mothur (v.1.47.0) following the MiSeq SOP pipeline (Schloss et al., 2009). This included filtering and the identification and removal of chimeras using vsearch (Rognes et al., 2016). After filtering sequences were taxonomically classified using the SILVA database (version 138) trained with 341F and 806R primers (Yu et al., 2005). ASV level results were classified. Amplicon sequence variants (ASVs) were then inferred and classified. ASVs present in fewer than 13 samples (50% of the total sample size, *n* = 26) were removed using the filter.shared() command in Mothur. Representative sequences of ASVs were obtained by using get.oturep() command in Mothur. Alpha diversity within hyphosphere samples was assessed in Mothur using Shannon diversity and Shannon evenness indices Spearman correlation analysis were calculated with the stats.spearmanr function from the SciPy Python package (Virtanen et al., 2020). Results of calculated correlation coefficients were visualized as a circular chord heatmap using matplotlib.pyplot python package. ASV abundance was also visualized using a chord diagram, generated with matplotlib.pyplot in Python. Beta diversity was evaluated using Principal Coordinate Analysis (PCoA), implemented via the PCoA function from the sklearn.decomposition module in Python.

#### 2.4.2 Bacterial isolate identification

To identify the isolates, colony PCR amplifications were carried out using the 27F (5’-AGAGTTTGATCMTGGCTCAG-3′) and 1492R (5’-GGTTACCTTGTTACGACTT-3′) (Lane, 1991) primers, and Sanger sequencing was carried out by Eton Biosciences. The resulting sequences were assembled to form full-length sequences. The resultant contigs were used to identify the bacterial isolate taxa by sequence match on the EzBiocloud database.

#### 2.4.3 Bacterial whole genome sequencing

From the initial collection of 37 hyphosphere-associated bacterial isolates, 10 representative strains were selected for whole genome sequencing based on 16S rRNA sequencing and BOX PCR fingerprinting using BOX-A1R primer 5′-CTACGGCAAGGCGACGCTGACG-3′. Strains were prioritized for sequencing based on their potential taxonomic diversity and putative functional relevance. Library preparation was performed using the Oxford Nanopore Technologies (ONT, United Kingdom) Native barcoding kit (SQK-NBD114.24), following the standard protocol. Sequencing was conducted on the Oxford Nanopore MinION platform. The prepared library was loaded onto the flow cell, and sequencing and basecalling were performed using MinKNOW (v23.10.4). Quality control of the raw reads was conducted using FastQC (v0.11.9) and NanoPlot (v1.41.0) to assess read length distribution, quality scores, and overall sequencing performance. Prior to assembly, reads were filtered using NanoFilt (v2.8.0) with a minimum quality threshold of Q10 and a minimum read length of 1,000 bp. *De novo* genome assembly was performed using Flye (v2.9.5), followed by polishing with Racon (v1.5.0) and Medaka (v1.11.3). Final assemblies were annotated with Prokka (v1.14.6). and assembly quality was evaluated using Quast (v5.2.0).

#### 2.4.4 Metabolic networks

To assess the metabolic potential of hyphosphere-associated bacterial isolates, Enzyme Commission (EC) numbers were extracted from the annotated genomes of the ten representative strains (R8, R5, M17, M5, BM01, M9, ACT01, W5, B2, and M24). These EC numbers were used to construct a genome-by-enzyme matrix reflecting the presence or absence and relative abundance of predicted enzymatic functions. This matrix was visualized as a clustered heatmap using the pheatmap package in R, with hierarchical clustering applied to both rows (EC numbers) and columns (genomes) using Euclidean distance and complete linkage. To provide a broader overview of enzymatic function, EC annotations were grouped into seven major enzyme classes: oxidoreductases, transferases, hydrolases, lyases, isomerases, ligases, and translocases. The number of EC annotations within each class was calculated per genome, and a bubble plot was generated using ggplot2, where bubble size represented annotation counts and color denoted enzyme class.

To gain pathway-level insight into microbial functional capabilities, metabolic network reconstructions were performed by manually mapping EC numbers to KEGG pathways using KEGG Mapper. For each pathway, enzymes were represented as color-coded bars indicating their presence or absence across the ten genomes, to compare conserved and variable metabolic capacities among isolates. The pathways analyzed included starch and sucrose metabolism, carbon fixation, methane metabolism, amino acid metabolism (phenylalanine, tyrosine, tryptophan), terpenoid backbone biosynthesis, folate biosynthesis, sulfur and nitrogen metabolism, polyketide sugar unit biosynthesis, and biosynthesis of various antibiotics.

### 2.5 Effect on fungal hyphal extension by the hyphosphere bacteria

Bacterial strains were cultured in tryptic soy broth (TSB) at 25 °C, shaking at 110 rpm for 48 h and diluted in TSB to an OD600 of 0.1 ± 0.03 approximating a 0.5 McFarland standard. Fungal strains were cultured in potato dextrose broth (PDB), harvested by centrifugation at 15,557 rcf and resuspended in phosphate-buffered saline (PBS) to be used as fungal inocula. For fungal hyphal extension analysis cocultures were plated on PDA with 0.3% agar with 10 μL of *P. laurylsulfatiphila* (M9) and 10 μL of FOV4 suspensions were spotted with a spatial separation of 4 cm across the diameter. Control plates with only bacteria or fungi were prepared using the same protocol. Imaging was conducted from day 3 to day 7. Hyphal extension was quantified using ImageJ software. The relative growth was expressed as the ratio [x/y], where x represents the hyphal extension of *Fusarium* in the presence of bacteria and y represents the corresponding growth in the absence of bacteria (control). This allowed for normalization across treatments and assessment of bacterial impact on fungal growth. Additionally, an isolate of *Fusarium oxysporum* f. sp. *vasinfectum* race 1 (FOV1), obtained from symptomatic cotton plants collected in Dawson County, Texas, was also included in the hyphal extension analysis.

## 3 Results

### 3.1 Microscopy

**Figure 1:**
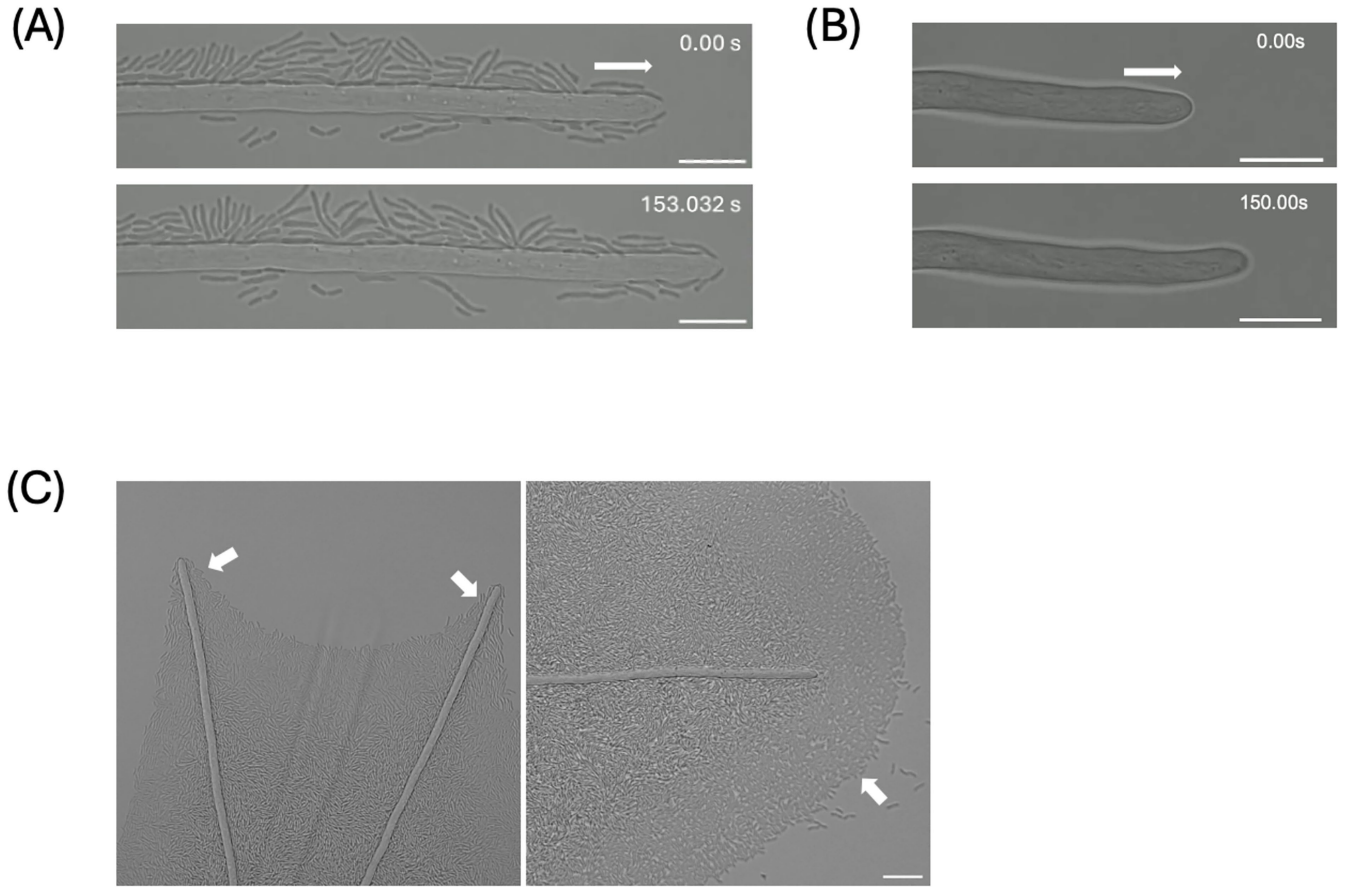
Presence and dispersal of bacterial pathobionts on the growing *Fusarium oxysporum* f. sp. *vasinfectum* Race 4 (FOV4) hyphosphere. **(A)** Time-lapse images [0–153 s] showing bacterial pathobionts associating with the growing hyphosphere of FOV4 and actively disperse to the hyphal tips [arrows – the direction of hyphal growth]. **(B)** Time-lapse images [0–150 s] of cured FOV4 hyphae demonstrating the absence of bacterial cells, confirming the specific association of pathobionts with untreated FOV4. **(C)** Hyphosphere-mediated bacterial dispersal observed along branching hyphae of FOV4 [arrows – bacterial movement surrounding the hyphae] Scale bar: 10 μm.

To demonstrate the physical interactions between the bacterial pathobionts and the hyphosphere of *Fusarium oxysporum* f. sp. *vasinfectum* Race 4 (FOV4), time-lapse microscopy was employed. In uncured fungal cultures, bacterial cells were observed associating with the surface of growing hyphae and migrating directionally towards the hyphal tips over time (Figure 1A). This dispersal pattern may potentially be guided by fungal exudates or surface structures. In contrast, time-lapse imaging of FOV4 hyphae that had been cured of associated bacteria showed a complete absence of bacterial cells along the hyphal surface (Figure 1B). The absence of bacterial cells in the cured hyphae confirms that the bacterial dispersal observed in uncured samples is specific and not due to contamination, thereby demonstrating a targeted physical interaction between bacterial pathobionts and fungal host. With confocal microscopy, we further demonstrated the localization of bacterial cells at hyphal branch points and surrounding the hyphal network (Figure 1C). Bacterial movement and accumulation appeared concentrated along actively growing regions of the hyphae, including tips and lateral branches, indicating that hyphal architecture may influence microbial positioning within the hyphosphere.

### 3.2 Metabarcoding

**Figure 2:**
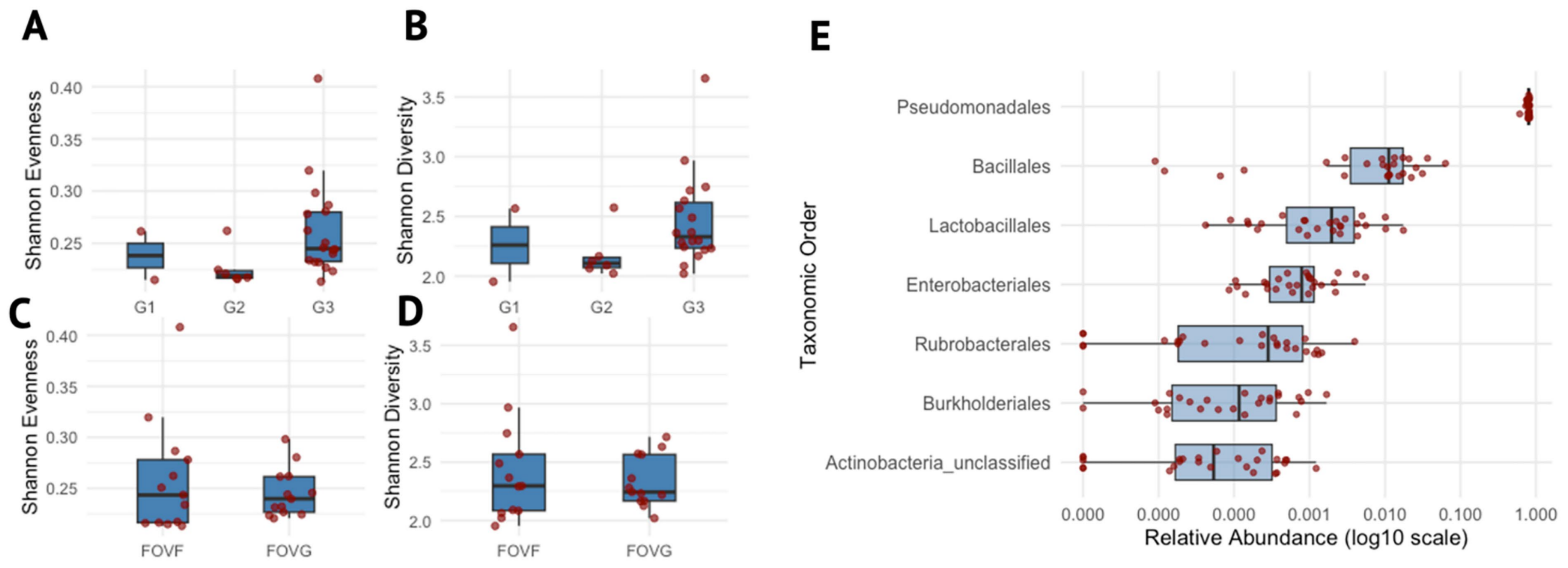
Microbial diversity and taxonomic distribution across generations and lineages. **(A–D)** denote box plots showing the distribution of Shannon evenness and Shannon diversity indices across different experimental groups. **(A,B)** Show diversity indices grouped by generations (G1- generation 1, G2 – generation 2, G3 – generation 3), whereas panels **(C,D)** show grouping by lineages (FOVF and FOVG). **(E)** Box plot depicting the relative abundance of taxonomic orders, only for those present in ≥75% of samples. Each order is shown on the y-axis, ordered by its median relative abundance, with the most abundant orders at the top. The x-axis represents the log10-transformed relative abundance. Data points are overlaid with jittered dots to represent individual values.

For hyphosphere samples, 2,256,763 sequences were recovered after quality filtering, representing a total of 335,684 ASVs identified following the MiSeq SOP with Mothur. At the phylum level, Proteobacteria were overwhelmingly dominant, accounting for 96.77% of all sequences. Other abundant taxa included Firmicutes (2.54%) and Actinobacteria (0.42%). The filter.shared() command retained only the ASVs that were present in at least 50% of the samples, and in the process removed the singletons and low-abundant ASVs, resulting in 54 ASVs. Alpha diversity was assessed across generations and lineages of FOV4 hyphospheres using Shannon diversity and Shannon evenness, revealing no differences among generations of isolates (Figures 2A–D). Pseudomonadales dominated the hyphosphere bacterial community at the order level across all samples. Beta diversity among hyphosphere samples and across hyphosphere generations was assessed and visualized using a 3D Principal Coordinates Analysis (PCoA) plot (Supplementary Figures 1A,B). The samples from different generations did not form distinct clusters but were instead intermixed, indicating no significant differences in community composition based on generation or sample replicate. This indicates a consistent hyphosphere microbiota community across generations, potentially structured by a single dominant taxon (Figure 2E).

**Figure 3:**
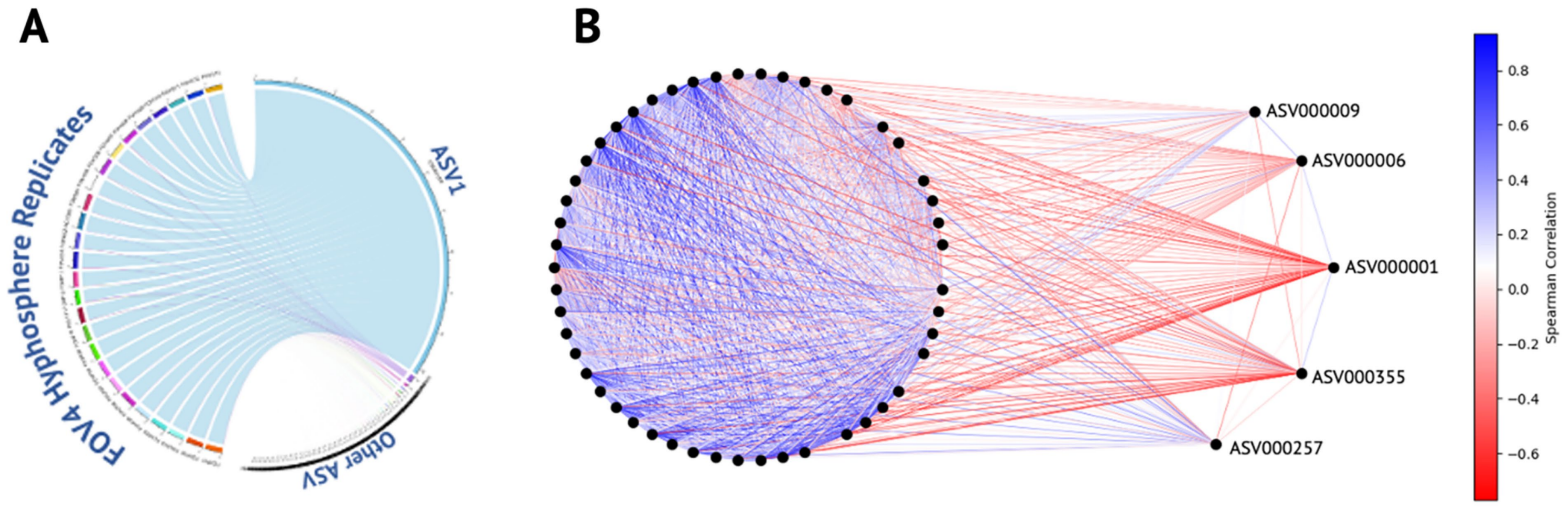
ASV distribution and correlation dynamics within the FOV4 hyphosphere. **(A)** Circular chord diagram illustrating the distribution of amplicon sequence variants (ASVs) across FOV4 hyphosphere replicates. ASV1, the dominant variant detected in all samples, is represented as the most prominent segment with strong links to each replicate, reflecting its high relative abundance and consistency across conditions. **(B)** Correlation network analysis of ASVs, where nodes represent individual ASVs and edges denote pairwise correlations. Edge color intensity indicates correlation direction and strength: darker blue represents strong positive correlations, while darker red indicates strong negative correlations. ASV1 is centrally positioned and displays strong negative correlations with the majority of ASVs, except ASVs 6, 9, 257, and 355, which remain unlinked by negative edges. The network highlights the distinct ecological positioning of ASV1 within the FOV4 hyphosphere community.

All samples were dominated by single ASV1 (*Pseudomonadaceae Pseudomonas*) which accounted for 96.72% of the total community composition, as illustrated in Figure 3A. Spearman correlation revealed a distinct community structure centered around ASV1, which exhibited predominantly negative correlations with most other ASVs in the dataset (Figure 3B; Supplementary Figure 2). Other ASVs with strong associations also included ASV6 (*Pseudomonadaceae Pseudomonas*), ASV9 (*Pseudomonadaceae Pseudomonas*), ASV000355 (*Listeriaceae Brochothrix*), and ASV000257 (*Enterobacteriaceae Enterobacter*) (Figure 3A). The most abundant ASVs (i.e ASV1, ASV6, and ASV9) within the Spearman correlation analysis were all identified as *Pseudomonadaceae Pseudomonas* and may represent closely related strains, or a consortium derived from a single *Pseudomonas* species. To evaluate their similarity, representative sequences from each ASV were aligned and compared. All representative sequences were 429 bases in length and differed by only five nucleotide positions, indicating a high degree of sequence similarity supporting the hypothesis that these ASVs may derive from the same organism. Moreover, the consistent presence of these closely related ASVs across two different isolates and three successive generations suggests a potential competitive advantage or selective interaction with the surrounding microbiota within the hyphosphere of FOV4.

### 3.3 Isolates numbers and descriptions

A total of 37 isolates were obtained as pure cultures, which include 13 strains from McConkey agar, 7 from yeast-extract mannitol agar, 5 from water yeast extract agar, and one isolate from BM agar. Direct heat treatment yielded 10 smooth bacillus-like colonies, while no actinomycete-like colonies were seen on tryptic soy agar or oatmeal agar. The enrichment followed by heat treatment produced bacillus-like colonies on tryptic soy agar, and streptomycete-like grey powdery colonies on oatmeal agar. One streptomycete-like colony was isolated as a pure culture.

### 3.4 Genomic characteristics and statistics

To assess the genomic diversity of hyphosphere-associated bacterial isolates recovered from *Fusarium oxysporum* f. sp. *vasinfectum* Race 4 (FOV4), whole-genome sequencing and *de novo* assembly were performed for ten representative strains spanning five bacterial genera: *Streptomyces, Microbacterium, Micrococcus, Pseudomonas*, and *Priestia*. A summary of key genomic features is presented in Table 1.

**Table 1:** Genomic characteristics of hyphosphere-associated bacterial species including *Streptomyces albogriseolus*, *Microbacterium algeriense*, *Micrococcus luteus*, *Pseudomonas laurylsulfatiphila*, and *Priestia megaterium*.

Assembly	# contigs (> = 1,000 bp)	Largest contig	Total length	GC (%)	N50	N90	L50	L90
*Streptomyces albogriseolus* (Act01)	76	1,635,525	7,535,416	72.57	348,604	174,394	5	36
*Microbacterium algeriense* (B2)	2	3,810,420	3,813,983	69.26	3,810,420	3,810,420	1	1
*Micrococcus luteus* (M24)	1	2,550,503	2,550,503	73	2,550,503	2,550,503	1	1
*Pseudomonas laurylsulfatiphila* (BM01)	66	1,050,635	6,776,523	60.08	226,758	43,593	8	32
*Pseudomonas laurylsulfatiphila* (M5)	10	1,593,175	6,824,038	60.09	926,600	518,057	3	7
*Pseudomonas laurylsulfatiphila* (M9)	15	1,615,535	6,611,667	60.08	875,345	320,449	3	7
*Pseudomonas laurylsulfatiphila* (M17)	25	1,408,730	6,849,035	60.09	656,456	199,805	4	11
*Pseudomonas laurylsulfatiphila* (R5)	12	1,390,133	6,821,084	60.09	978,934	319,147	3	7
*Pseudomonas laurylsulfatiphila* (R8)	30	879,017	6,892,494	60.08	494,017	158,295	6	16
*Priestia megaterium* (W5)	26	5,028,496	5,696,661	38.05	5,028,496	5,028,496	1	1

The table includes number of contigs, contig size, genome size, GC content, and additional genomic features (including N50 and N90 represent the contig lengths such that 50 and 90% of the total assembly length are contained in contigs of equal or greater length, respectively. L50 and L90 indicate the minimum number of contigs required to cover 50 and 90% of the total assembly length, respectively. Higher N-values and lower L-values reflect greater assembly contiguity).

The genome sizes of the ten bacterial isolates ranged from 2.55 Mb (*Micrococcus luteus* M24) to 7.54 Mb (*Streptomyces albogriseolus* Act01), with a mean genome length of 6.01 Mb. Members of the Actinobacteria (*Streptomyces, Micrococcus, Microbacterium*) displayed larger genome sizes and high GC content, consistent with their known metabolic versatility and complex life cycles. In contrast, the genome of *Priestia megaterium* W5 was relatively compact (5.7 Mb), exhibiting the lowest GC content in the dataset (38.05%) but demonstrating high assembly contiguity.

Among the six *Pseudomonas laurylsulfatiphila* isolates, genome sizes were tightly clustered between 6.61 and 6.89 Mb, indicating high genomic conservation within the group. However, assembly contiguity varied substantially, with contig counts ranging from 10 (M5) to 66 (BM01), likely reflecting differences in sequencing depth, DNA quality, or repetitive content. Despite similar genome sizes and GC content, *P. laurylsulfatiphila* isolates exhibited marked variation in contiguity. The largest contigs ranged from 879,017 bp in R8 to 1,615,535 bp in M9, with N50 values from 226,758 bp (BM01) to 978,934 bp (R5) and L50 values between 3 and 8.

The most contiguous assemblies were observed for *M. luteus* M24 and *M. algeriense* B2, each assembled into one or two contigs, with N50 values equal to their total genome lengths (2.55 Mb and 3.81 Mb, respectively), suggesting near-complete circular chromosomes. *P. megaterium* W5 also showed a highly contiguous assembly (N50 = 5.03 Mb; L50 = 1). In contrast, the genome of *S. albogriseolus* Act01 was more fragmented, consisting of 76 contigs with an N50 of 348.6 kb, which is consistent with the known assembly challenges associated with high-GC, linear actinomycete genomes. GC content across the dataset ranged from 38.05 to 73.00% and generally followed phylogenetic patterns. Actinobacterial genomes had the highest GC content (*S. albogriseolus*, 72.57%; *M. algeriense*, 69.26%; *M. luteus*, 73.00%), while *Pseudomonas* genomes were relatively uniform (60.08–60.09%), and *P. megaterium* exhibited the lowest GC content (38.05%), characteristic of Bacillaceae.

### 3.5 Metabolic networks and functional profiling of hyphosphere bacteria

**Figure 4:**
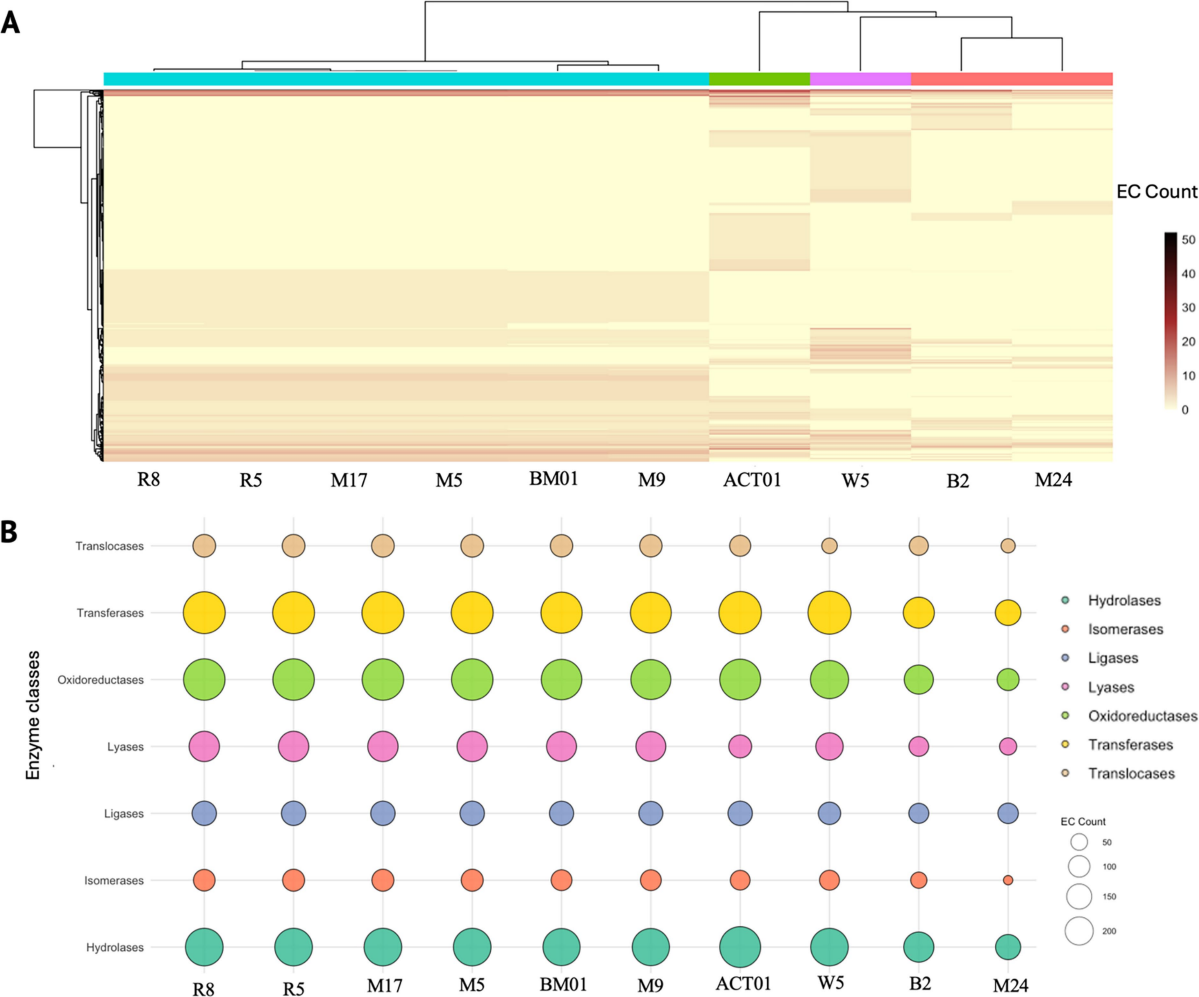
Functional enzyme distribution across hyphosphere-derived bacterial genomes based on enzyme commission (EC) classification. **(A)** Heatmap showing the abundance of predicted enzyme functions across ten bacterial genomes isolated from the hyphosphere of FOV4. Each column represents a genome (*Pseudomonas laurylsulfatiphila* – [R8, R5, M17, M5, BM01, M9], *Streptomyces albogriseolus* – ACT01, *Priestia megaterium* – W5, *Microbacterium algeriense* – B2, *Micrococcus luteus* – M24), and each row corresponds to a unique EC number. Hierarchical clustering reveals functionally distinct profiles among bacterial isolates. **(B)** Bubble plot illustrating the distribution of major enzyme classes; hydrolases, isomerases, ligases, lyases, oxidoreductases, transferases, and translocases across the same genomes (bubble size – number of EC annotations per enzyme class, and color – enzyme type).

To evaluate the metabolic potential of hyphosphere-associated bacterial isolates, the enzyme commission (EC) numbers were extracted from the annotated genomes of the 10 selected bacterial isolates described in Table 1 for whole-genome sequencing. These EC numbers were used to generate a genome-by-enzyme matrix reflecting the presence and abundance of predicted enzymatic functions. Hierarchical clustering of this matrix revealed clear variation in enzyme distribution across genomes (Figure 4A). Isolates of *Pseudomonas laurylsulfatiphila* exhibited broad and dense EC profiles, while *Streptomyces albogriseolus* (ACT01) and *Micrococcus luteus* (M24) displayed more restricted enzyme repertoires, suggesting differential metabolic capacity among isolates.

Classification of EC numbers into seven major enzyme classes: oxidoreductases, transferases, hydrolases, lyases, ligases, isomerases, and translocases, enabled comparison of functional distributions across isolates (Figure 4B). Oxidoreductases and transferases were the most abundant enzyme classes across all genomes. Several *P. laurylsulfatiphila* strains showed increased counts of hydrolases and oxidoreductases compared to other taxa. To place these enzyme functions within broader metabolic frameworks, EC numbers were manually mapped to KEGG pathways using KEGG Mapper. Reconstructed pathways included both conserved and variable metabolic processes (Supplementary Figures S3A–J). Carbohydrate metabolism pathways, such as starch and sucrose metabolism, were widely represented, with key enzymes including starch synthase, *α*-amylase, and phosphoglucomutase detected across multiple isolates. Enzymes associated with carbon fixation pathways, such as phosphoenolpyruvate carboxylase and malate dehydrogenase, were present in several genomes. Intermediates shared with the Calvin cycle and pentose phosphate pathway, such as glyceraldehyde-3-phosphate and sedoheptulose-7-phosphate, were also identified.

Genes involved in methane metabolism were detected in several isolates, including those coding for enzymes linked to intermediates like glycerate, oxaloacetate, and acetyl-CoA. While complete methylotrophic pathways were not recovered, individual enzymes overlapped with glycolytic and TCA cycle processes. Broad conservation of amino acid biosynthetic pathways was observed, particularly for aromatic amino acids (e.g., phenylalanine, tryptophan, tyrosine). Shikimate pathway enzymes, including chorismate synthase, were identified across several isolates. Multiple genomes encoded enzymes involved in terpenoid backbone and folate biosynthesis, such as 1-deoxy-D-xylulose-5-phosphate synthase and dihydrofolate synthase. Sulfur metabolism genes, including adenylyl sulfate reductase and cysteine synthase, were also detected. Genes involved in nitrogen metabolism, including nitrate reductase, nitrite reductase, and glutamine synthetase, were present in most genomes, suggesting the presence of assimilatory nitrate reduction and ammonia assimilation potential.

Overall, the functional annotation and pathway reconstruction reveal a broad distribution of metabolic functions among the hyphosphere-associated bacterial isolates. While core pathways related to carbon, nitrogen, and sulfur metabolism were conserved, variation in secondary and cofactor biosynthetic pathways was observed between genera, indicating potential metabolic differentiation among the FOV4 associated bacterial taxa.

### 3.6 Hyphal extension

**Figure 5:**
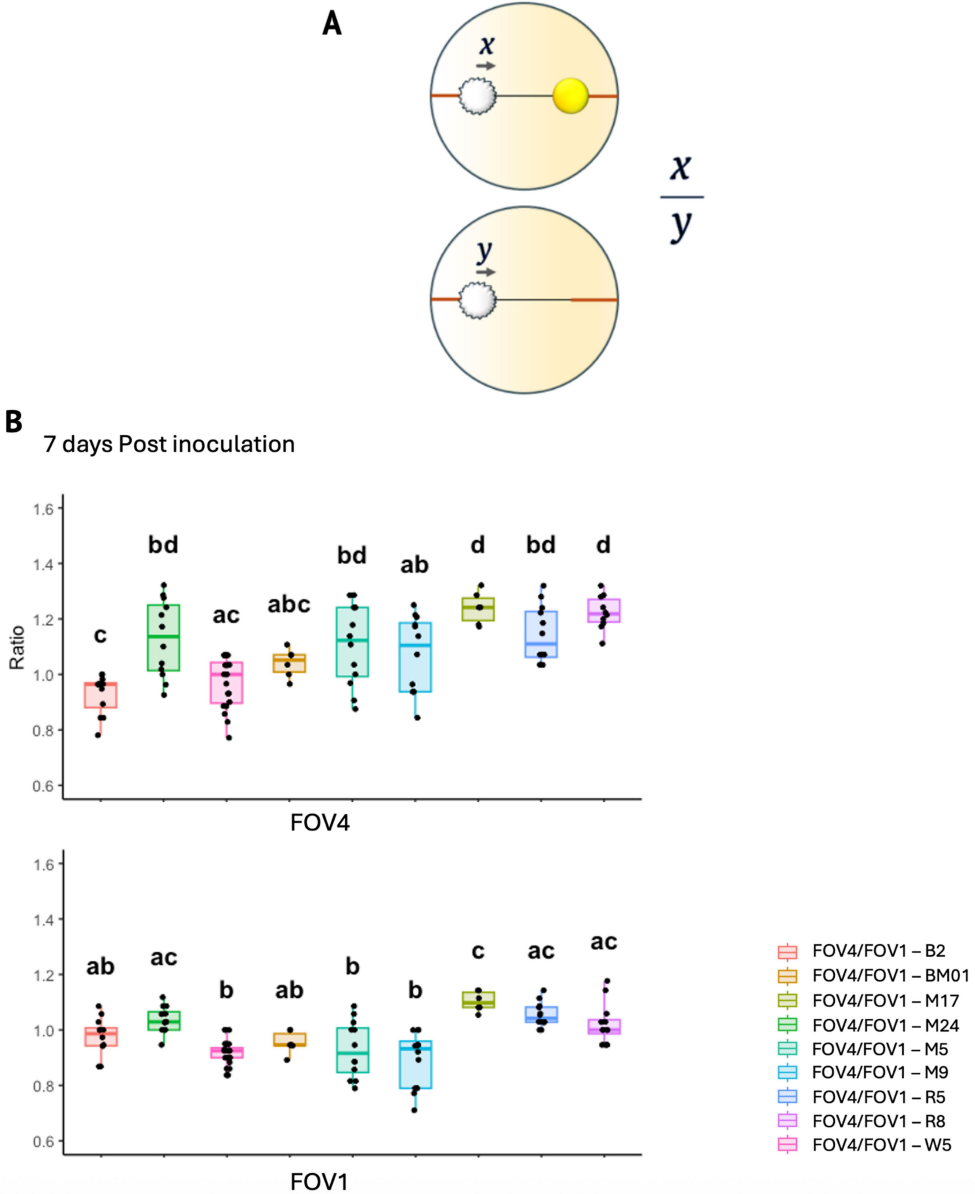
Effect of bacterial candidates on FOV4 and FOV1 hyphal extension. **(A)** Schematic representation of the hyphal growth ratio measurement used in the assay. The ratio [x/y] represents hyphal extension of *Fusarium* in the presence of bacteria (x) relative to its growth in the absence of bacteria (y). **(B)** Boxplots showing the effect of hyphosphere bacterial isolates on hyphal extension of FOV4 [top] and FOV1 [bottom] at 7 days post-inoculation. Each treatment corresponds to a co-culture of FOV4 or FOV1 with a specific bacterial strain, as indicated in the legend (*p* < 0.05, each dot represents an individual biological replicate).

Given that the hyphosphere bacteria possess diverse metabolic functions, including pathways involved in nutrient acquisition, Reactive oxygen species (ROS) modulation, and signaling compound biosynthesis, we sought to determine whether these bacterial isolates could influence fungal growth. Some of the bacteria originally isolated from the hyphosphere of FOV4 may promote its hyphal extension, potentially reflecting a cooperative or facilitative interaction that could enhance pathogenic fitness. To evaluate this influence of hyphosphere bacteria on fungal growth dynamics, we conducted coculture assays measuring hyphal extension of FOV4 and FOV1 in the presence of each bacterial strain. The assay utilized a growth ratio index (x/y), where x represents hyphal extension in the presence of bacteria and y corresponds to growth in the absence of bacterial interaction (Figure 5A). Ratios greater than 1 indicate bacterial-promoted fungal growth, while ratios below 1 suggest inhibition.

At 7 days post-inoculation (Figure 5B), several bacterial strains significantly influenced FOV4 hyphal extension. Cocultures with *Microbacterium algeriense* (B2) showed an inhibitory effect, while strains such as *Micrococcus luteus* (M24), *Pseudomonas laurylsulfatiphila* (R5, M9), and *Priestia megaterium* (W5) consistently promoted FOV4 hyphal growth (ratio > 1.2). Notably, the same bacterial strains exhibited inconsistent effects on FOV1 hyphal extension, suggesting differential fungal responses to microbial stimuli between races.

To capture temporal dynamics, fungal-bacterial cocultures were also monitored at earlier time points (3 and 5 days post-inoculation) as shown in Supplementary Figure 4. At 3 days post-inoculation (Supplementary Figure S4A), significant stimulation of FOV4 growth was observed for *P. laurylsulfatiphila* strains M17, M5, and R8, whereas the impact on FOV1 remained minimal or neutral. By day 5 (Supplementary Figure S4B), bacterial effects on FOV4 began to stabilize, with several strains (e.g., BM01, M24, M5) maintaining a growth-promoting trend, while B2 continued to exhibit inhibitory influence. In contrast, FOV1 responses remained variable across timepoints, with no single strain showing consistent promotion or suppression of growth. These results demonstrate that several hyphosphere bacterial isolates can modulate hyphal extension of FOV4, with more limited or variable effects observed on FOV1.

## 4 Discussion

The hyphosphere, defined as the microhabitat surrounding fungal hyphae, is a dynamic zone of microbial interaction that can significantly influence fungal biology (Sun et al., 2023; Pugh and Sewell, 1958; Frey-Klett and Garbaye, 2005; Frey-Klett et al., 2011). While endohyphal bacterial association with plant pathogenic fungi has been documented, extrahyphal interactions remain underexplored. Building on recent findings from our lab, documenting bacterial associations in the hyphosphere of pathogenic fungi (Thomas and Antony-Babu, 2024), we extend this concept to *Fusarium oxysporum* f. sp. *vasinfectum* Race 4 (FOV4), a major pathogen threatening cotton in Texas. Using the pathobiome framework, we investigate the extrahyphal bacterial associations of FOV4 to better understand their spatial dynamics, genetic potential, and ecological significance.

In this study we used three different approaches towards demonstrating and defining the FOV4 hyphosphere bacteriome. We visually demonstrate the presence of bacteria through confocal microscopy (Figure 1), culture-independent assessment (Figures 2, 3), and culture-dependent methods. Time-lapse microscopy revealed bacterial accumulation at the hyphal tips of FOV4, suggesting a directed interaction potentially mediated by metabolites, consistent with previous work demonstrating that specific metabolites at hyphal apices can mediate bacterial fungal mutualisms, including thiamine exchange between fungal mycelia and associated bacteria (Abeysinghe et al., 2020). In our ongoing work, MALDI-TOF imaging has revealed spatial differentiation in metabolite profiles across fungal colonies, with hyphal tips exhibiting distinct chemical signatures compared to older hyphal regions (unpublished data). These findings align with previous studies showing that hyphal exudates influence bacterial localization in soil microhabitats (Zhou et al., 2020), and exhibit higher metabolic activity (Guhr et al., 2015; Yasuda et al., 2021). As the hyphal tip is also the primary point of contact with the rhizosphere and plant roots, its role in microbial recruitment is ecologically significant. Similar bacterial accumulation at hyphal tips was previously documented in our laboratory (Thomas and Antony-Babu, 2024), further substantiating the spatial specificity of these fungal-bacterial interactions. In future work, this hypothesis could be experimentally tested using chemotaxis assays with FOV4 tip-conditioned medium in microfluidic or agarose-gradient systems, coupled with chemical perturbation of candidate exudates. Additionally, time-lapse imaging of antibiotic treated cultures showed normal hyphal extension and branching patterns comparable to untreated samples. These visual observations suggest that bacterial removal did not impair fungal viability, indicating that the associated bacteria are likely not obligate symbionts. However, these bacteria may still be recruited for other roles, such as enhancing virulence or providing trace nutrients.

The 16S rRNA gene based metabarcoding for bacterial diversity on the hyphosphere of FOV4 revealed a surprisingly stable microbial profile. Our original goal for sequential subculture was reduction of diversity with each generation; however, the hyphosphere was stable and largely unaltered. Alpha diversity did not change between the two lineages of subcultures, nor by the generations of subcultures (Figures 2A–D). We also noted a similar lack of change in community structure as shown in PCoA analysis (Supplementary Figure 2). Interestingly, the hyphosphere community was dominated by Pseudomonads (>95%; Figure 2E). This aligns with previous studies, where *Pseudomonas* spp. has been implicated in both plant health and disease management (Elhady et al., 2025). The consistent dominance of *Pseudomonas* in the FOV4 hyphosphere suggests that they may possess traits that confer a competitive or beneficial association with the fungus, though the nature of this relationship remains to be elucidated. However, there might be clues from earlier literature. For instance, Frey-Klett et al. (1997) and Deveau et al. (2007) showed that *Pseudomonas fluorescens* BBc6R8 serves as a fungal helper organism, assisting mycorrhizal fungal colonization of the roots through its Type III secretion system (Cusano et al., 2011).

Additionally, a single ASV assigned to the genus *Pseudomonas* dominated all the samples (Figure 3A), the ASV that interestingly showed negative correlation (Figure 3B; Supplementary Figure 1) with almost all other core ASVs. The exceptions were two other *Pseudomonas* ASVs and one ASV each assigned to *Bronchothrix* and *Enterobacter*. The correlation scores were low enough for *Bronchothrix* and *Enterobacter* that they might not have real correlation. However, the two other *Pseudomonas* ASVs that were positively correlated could be attributed to kin selection known between *Pseudomonas* strains (Belcher et al., 2022). However, this might be a case of intragenomic heterogeneity, which has also been demonstrated in *Pseudomonas,* where more than one ASV can be from the same *Pseudomonas* genome (Bodilis et al., 2012).

Three core bacterial taxa of the FOV4 hyphosphere, Pseudomonadales, Bacillales, and Actinobacteria, were successfully isolated using culture-dependent assays. Genomic profiling of bacterial isolates from the hyphosphere revealed a diverse array of metabolic pathways, including those involved in carbon, nitrogen, and sulfur metabolism. These findings suggest that bacteria in the hyphosphere may play crucial roles in nutrient cycling, potentially impacting the health of both the pathogen and host plant. Support for such positive fungal-bacterial associations largely stems from mycorrhizal systems, where pseudomonads and actinobacteria have been shown to enhance fungal nutrient acquisition. For instance, Pseudomonadales secrete enzymes like proteases, glucanases, and chitinases that facilitate carbon and nitrogen uptake by their fungal partners (Sah et al., 2021), while actinobacteria have been reported to improve nutrient assimilation in *Funneliformis mosseae* during mung bean and rice cultivation (Lasudee et al., 2018).

The most abundant enzyme categories across all genomes were oxidoreductases and transferases, with strains of *Pseudomonas laurylsulfatiphila* showing enrichment in hydrolase and oxidoreductase activities (Figure 4). Such patterns may reflect ecological adaptations related to carbon turnover (Tenorio-Salgado et al., 2024), redox balance (Borisov et al., 2021), and polymer degradation (Morisseau, 2022) in the hyphosphere. These enzymatic functions are particularly relevant in the context of host–microbe interactions, as oxidoreductases are known to modulate reactive oxygen species (ROS), while hydrolases can degrade host or pathogen-derived polymers, influencing colonization and nutrient dynamics (Zamioudis et al., 2013; Raaijmakers et al., 2009). Furthermore, differences in GC content among the hyphosphere-associated bacterial genomes may relate to functional specialization, including secondary metabolite biosynthesis. Future comparative analyses integrating pangenome profiling and secondary metabolite biosynthetic gene cluster prediction could clarify whether GC content variation is associated with specific functional capacities in these taxa.

Many of the KEGG pathways were found to exhibit redundancy, with key pathways such as sugar metabolism, carbon fixation, IAA synthesis, and nitrate reduction being carried out by multiple bacterial members (Supplementary Figures 3A–J). Previous studies have suggested that functional redundancy in the hyphosphere core microbiome may contribute to community stabilization (Wang et al., 2024), especially in enzymes that degrade and synthesize key substrates (Brandt et al., 2025). This redundancy acts as a form of ‘insurance’ against perturbations (Ramond et al., 2024), potentially mitigating the challenges posed by pathogens in response to plant defense mechanisms. Of particular interest is the observation that many of the core functions identified are associated with plant growth promotion. Although compounds such as IAA auxins are often linked to pathogenic processes, it is plausible that the microorganisms involved in the pathogen’s hyphosphere may also serve as plant growth promoters typically found in the rhizosphere. Our findings suggest that pathogens may exploit plant-beneficial microbes, recruiting them from the existing rhizosphere microbiota to enhance their own colonization and survival (Carrión et al., 2019). It is therefore reasonable to hypothesize that pathogens capable of manipulating or co-opting the host’s microbial network gain a competitive advantage in terms of virulence. We believe that this question whether plant-beneficial microorganisms collaborate with the pathogens warrants further investigation.

Our coculture assays demonstrated that bacterial strains, including *Pseudomonas laurylsulfatiphila*, significantly influence fungal growth, as evidenced by the consistent promotion of hyphal extension in *Fusarium oxysporum* f. sp. *vasinfectum* (FOV4). This suggests that these bacteria may enhance fungal fitness through metabolic interactions. Notably, such effects were not observed when the same bacterial strains were cocultured with a different strain of *F. oxysporum*. This strain-specific interaction warrants further investigation to better understand the dynamics of bacterial influence on fungal growth in natural ecosystems. A strain-specific hyphosphere community was also suggested by Thomas and Antony-Babu (2024). The finding that the interactions between pathogens and their associated pathobionts are specific could serve as a potential target for disease control and management strategies. Further studies into the underexplored hyphosphere-pathobiome interactions are essential to elucidate these complex microbial relationships and their implications for plant health and disease management.

Furthermore, our study demonstrates that a stable bacterial community is consistently associated with the hyphae of a potent Pima cotton pathogen. Based on the data presented, we propose that pathobiont bacteria and pathogenic fungi may function independently under certain conditions, but can interact and form a hyphosphere-pathobiome under specific, yet undetermined, circumstances. In the case of FOV4, Davis et al. (2022) reported an uneven spatial distribution of the pathogen in cotton fields affected by FOV4, a pattern that did not correlate with disease symptoms. We suggest that these field-scale variations may also result from differences in microbial communities, particularly those associated with the hyphae. Given that both the bacteria and fungi can be cultivated separately, it is plausible that the pathobiome-bacteria association could be gained or lost, influencing the virulence of the pathogen. While these speculations regarding the role of hyphosphere bacteria in FOV4 virulence require further exploration, they underscore the need for deeper investigation into the dynamics of the hyphosphere-pathobiome.

In summary, our investigation into the hyphosphere of *Fusarium oxysporum* f. sp. *vasinfectum* Race 4 (FOV4) highlights the dynamic interplay between pathogenic fungi and associated bacterial communities. Through advanced microscopy and genomic analyses, we have uncovered a stable, yet diverse bacterial consortium dominated by *Pseudomonas* spp. These bacteria, enriched with diverse enzymatic capabilities, likely contribute to nutrient cycling and fungal fitness within the hyphosphere. Our findings underscore the potential of these microbial interactions in influencing pathogen virulence and plant health. Further exploration of these complex hyphosphere-pathobiome dynamics is crucial for developing targeted strategies in agricultural disease management and enhancing crop resilience.

## 5 Conclusion

This study provides novel insight into the hyphosphere of *Fusarium oxysporum* f. sp. *vasinfectum* Race 4 (FOV4), revealing a stable and functionally diverse bacterial community dominated by *Pseudomonas* spp. Integrating microscopy, metabarcoding, and genomic profiling, we demonstrate that these bacterial associates are spatially enriched at fungal hyphal tips and possess metabolic traits potentially contributing to nutrient cycling and fungal fitness. Despite being nonobligate symbionts, these bacteria may modulate fungal growth and virulence in a strain-specific manner, reinforcing the pathobiome framework as a valuable lens for interpreting fungal bacterial interactions.

Our findings highlight the ecological and functional relevance of extrahyphal bacterial associations and suggest that the FOV4 hyphosphere may serve as a microbial interface through which pathogens recruit or co-opt rhizosphere microorganisms. This interaction may contribute to pathogen success *in planta*, with potential implications for disease progression and field-level variability. Future research into the specificity and dynamics of hyphosphere-pathobiome interactions will be critical for developing microbiome-informed strategies to manage soilborne fungal diseases and enhance crop resilience.

## Data Availability

All sequencing data generated in this study have been deposited in the NCBI database under BioProject accession number PRJNA1180603. This includes 16S rRNA gene amplicon sequences from FOV4 hyphosphere metabarcoding (SRX26599052–SRX26599077) and whole-genome sequencing data for ten bacterial isolates (SRX29083462–SRX29083471).
